# Acute effects of regional heat stimulation by indirect moxibustion on cardiovascular responses

**DOI:** 10.1186/s12576-022-00855-z

**Published:** 2022-11-24

**Authors:** Hidehiro Nakahara, Eriko Kawai, Tadayoshi Miyamoto

**Affiliations:** 1 Graduate School of Health Sciences, Morinomiya University of Medical Sciences, 1-26-16 Nankokita, Suminoe, Osaka, 559-8611 Japan; 2 RIKEN Center for Biosystems Dynamics Research Laboratory for, Pathophysiological and Health Science, 6-7-3 Minatojima-Minamimachi, Chuo-Ku, Kobe, Hyogo 650-0047 Japan; 3 Department of Sport and Health Sciences, Faculty of Sport and Health Sciences, Osaka Sangyo University, Wellness 2008, 3-1-1, Nakagaito, Daito, Osaka 573-1004 Japan

**Keywords:** Heart rate, Blood pressure, Skin temperature, Moxibustion, Heat stimulation

## Abstract

Moxibustion is a traditional East Asian medicine treatment that involves burning moxa directly or indirectly on or near the skin at a specific site of the body, called an acupoint. However, whether moxibustion induces cardiovascular responses by modulating autonomic nervous activity remains unknown. The purpose of this study was to elucidate the effects of indirect moxibustion on cardiovascular responses and autonomic nervous activity. Fifteen healthy volunteers participated in the study. Each subject received regional heat stimulation by indirect moxibustion at the lower leg acupoint. Heart rate, RR intervals, blood pressure and skin temperature were measured continuously for 3 min at rest and 5 min during indirect moxibustion. Local skin temperature increased reaching a peak (45.3 ± 3.3 °C) at 2 min after moxibustion was started, and was accompanied by a significant decrease in heart rate (63.0 ± 7.8 to 60.8 ± 7.8 bpm, *p* < 0.05) together with a significant increase in root mean square difference of successive RR intervals. Regional heat stimulation by indirect moxibustion induced bradycardic response, which was modulated by autonomic nervous system.

## Introduction

Moxibustion is an alternative medicine treatment that involves burning moxa on or near the skin at a specific site of the body, called the acupoint. Moxibustion has been promoted as a treatment for pain and gastrointestinal problems such as irritable bowel syndrome [1, 2]. In clinical practice, moxibustion has been used to treat cardiovascular diseases including hypertension, but this use is not supported by research evidence. Indirect moxibustion is a gentle form of moxa that uses a buffer substance such as paper pulp, ginger, garlic or salt between the heated moxa and the skin to avoid direct contact, and is highly safe and versatile. Shu et al. [3] studied patients with chronic fatigue syndrome, and found that indirect moxibustion induced improvement of the Fatigue Assessment Instrument score after the 4th and 10th treatments. Based on the results of heart rate variability (HRV) indices, the possible mechanism of the observed effect may involve activation of the vagal nerve. However, the extent to which cardiovascular responses are modulated by indirect moxibustion during the short duration of heat stimulation (acute effects) is yet to be elucidated.

Previous studies demonstrated that thermotherapy using hot packs or heat- and steam-generating sheets significantly decreased mean arterial blood pressure [4] and heart rate, and increased parasympathetic nervous activity [5, 6]. Indeed, Yasui et al. [5] showed that the heat- and steam-generating sheet induced a decrease in heart rate accompanied by increases in parasympathetic nervous activity and fatigue perception compared to the control sheet. They suggested that the application of heat stimulation to the neck region induces a relaxing effect associated with an increase in parasympathetic nervous activity. Nagashima et al. [6] also reported that application of the heat- and steam-generating sheet to the lumbar or abdominal region increased RR interval and parasympathetic nervous activity. Our preliminary study observed that constant and/or acute heat stimulation at the lower leg induced bradycardic response [7]. Therefore, we hypothesized that heat stimulation by indirect moxibustion at the lower leg may induce a decrease of heart rate elicited by increased parasympathetic nervous activity.

Moxibustion is usually applied to acupoints, which are small areas in the body. The duration of heat stimulation by indirect moxibustion is also rather short compared to the that provided by thermotherapy using hot packs or heat- and steam-generating sheets. Therefore, moxibustion has advantage in clinical application because it efficiently stimulates smaller regions within a short time compared to previously studied techniques. The purpose of this study was to investigate the effects of indirect moxibustion on cardiovascular responses and autonomic nervous activity.

## Methods

### Subjects

Fifteen volunteers comprising 11 males and 4 females aged 20.8 ± 0.4 (mean ± SD) years participated in the present study. The subjects were asked to refrain from consuming caffeine and alcohol, and to avoid strenuous exercise for 24 h prior to the experiment. All protocols were reviewed and approved by the Ethics Committee of Morinomiya University of Medical Sciences (No. 2019-057). Written informed consent in accordance with the Declaration of Helsinki was obtained from all subjects after they were given full explanations of the objectives, methods, and potential risks of the study.

### Indirect moxibustion

The indirect moxibustion apparatus (Sennenkyu Off Ibuki, Senefa, Shiga, Japan) consisted of a paper tube (10 mm in height, 5 mm in diameter) filled with moxa and a buffer substance (3 mm in height, 15 mm in diameter). Paper pulp was used as the buffer substance between moxa and skin to avoid direct contact of heat with skin. Indirect moxibustion was performed at the acupoint of Zusanli (WHO; ST36) overlying the deep peroneal nerve and tibialis anterior muscle on the right side. The reason for using this acupoint is that ST36 is an important acupoint that modulates autonomic nervous activities [8].

### Experimental tasks

The experiment was designed to investigate the effect of indirect moxibustion on heart rate and blood pressure responses. Subjects were requested to rest in a supine position for approximately 5 min. Then, disposable electrocardiogram electrodes, blood pressure measurement device, and thermometer were attached. After baseline blood pressure and heart rate were recorded for 3 min under resting condition, two experimental tasks were implemented: indirect moxibustion with burning moxa was performed for 5 min (heat stimulation task), and indirect moxibustion with non-burning moxa was performed for 5 min (control task). The subjects underwent both tasks in a random order. The two tasks were tested on separate days with a 7 day interval.

### Data analysis

Heart rate was monitored continuously with a three-lead electrocardiogram telemeter (BSM-7201, Nihon Kohden Co., Tokyo, Japan). Blood pressure was recorded continuously in the left arm using an automatic blood pressure monitoring device with tonometry (BP-608, Omron Colin Co., Ltd, Tokyo, Japan). This blood pressure monitoring device utilizes a state-of-the art multisensor array technology to detect pulse waves at the radial artery. Before each trial, the device was calibrated using arterial pulse waves obtained by the oscillometric method. Skin temperature was monitored continuously with a digital thermometer (PTC-401, Unique Medical, Osaka, Japan). The sensor of the thermometer was placed at the center of the buffer substance. Heart rate, blood pressure, and skin temperature were sampled at 200 Hz and stored on a laboratory computer system. Heart rate, blood pressure, and skin temperature values were averaged in 60 s segments in each subject. The values averaged over the last 60 s of the 3 min rest period were used as the data for resting condition, and the values averaged over the 60 s between 300 and 360 s when the skin temperature reached a peak were used as the data for moxibustion condition.

### Time-varying vagal-related heart rate variability measurement

**Fig. 1:**
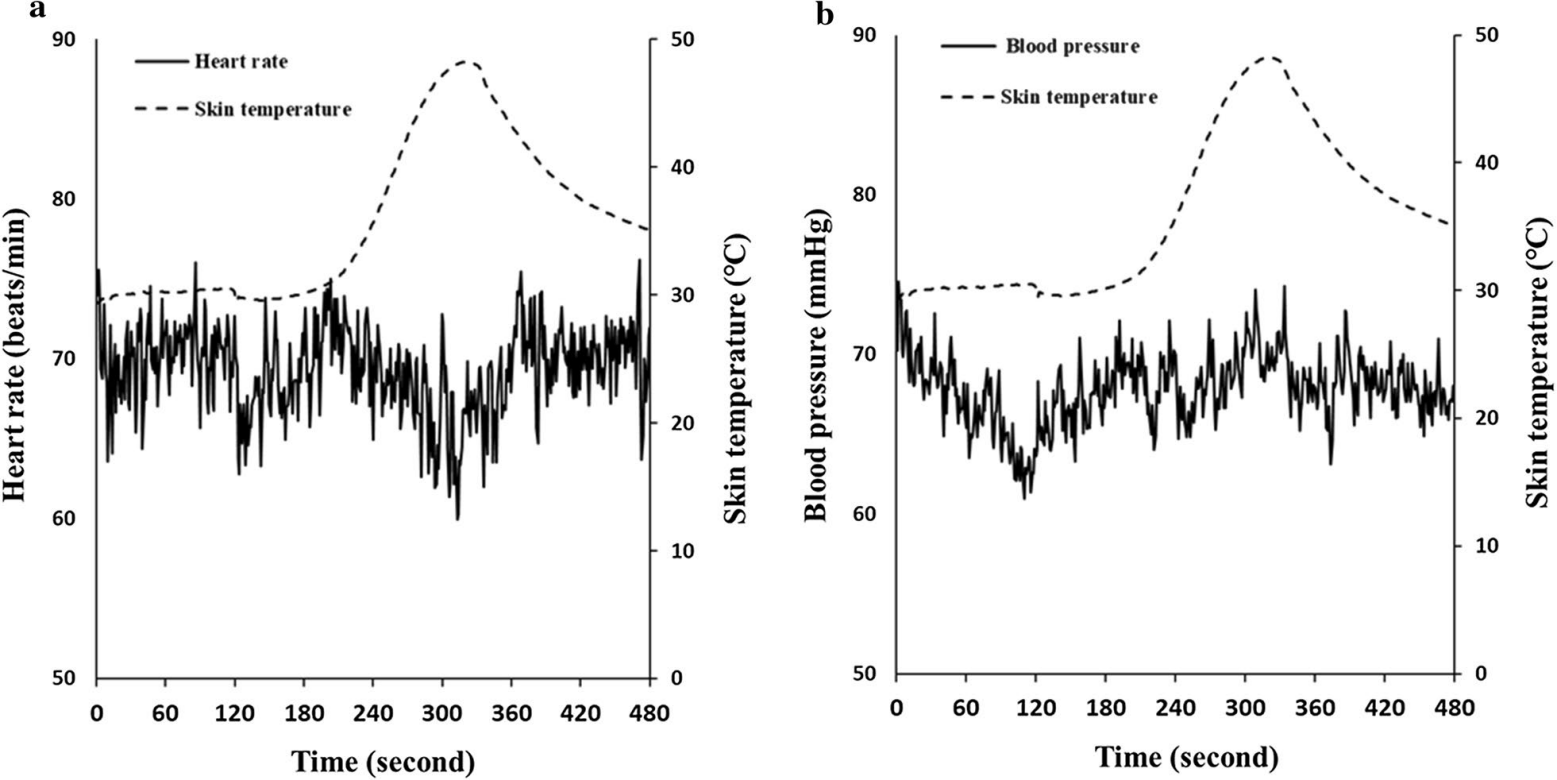
Representative traces of heart rate (**a**) and blood pressure (**b**) responses to heat stimulation by indirect moxibustion in the same patient. The solid line represents heart rate or blood pressure, and the dotted line represents skin temperature

HRV is regulated by respiration and heat [9, 10]. Previous studies have suggested that ultra-short-term HRV can be estimated from the root mean square difference of successive normal RR intervals (RMSSD) [9, 10]. HR response induced by heat stimulation is also observed in the short period during indirect moxibustion (Fig. 1). Thus, we calculated RMSSD as a time-varying vagal-index for each condition [9, 10]. For analysis of HRV, RR interval was measured using a three-lead electrocardiogram in each subject. Artifacts of RR interval such as ectopic beats and missing beats were identified by visual inspection and deleted. RMSSD was calculated from the 60 s data for resting condition and moxibustion condition.

### Statistical analysis

The effects of task (non-burning moxa and burning moxa) and condition (resting condition and moxibustion condition) on each variable examined were tested statistically using two-way repeated measures analysis of variance (ANOVA). Post-hoc analysis was conducted using Tukey’s procedure. Simple correlation analysis was performed to identify the effect of heat stimulation by indirect moxibustion on cardiovascular responses over 8 min of the experimental period. For simple correlation analysis, the mean values per minute averaged over 8 min of the experimental period were used. Statistical significance was accepted at *p* < 0.05.

## Results

Figure. 1a shows representative traces of heart rate response and skin temperature recorded in one subject. Heart rate decreased as skin temperature increased. The decrease in heart rate was most marked at around 300 s when the skin temperature reached a peak. Figure. 1b depicts representative traces of blood pressure response and skin temperature recorded in the same subject. Blood pressure did not change with increase in skin temperature.

**Fig. 2:**
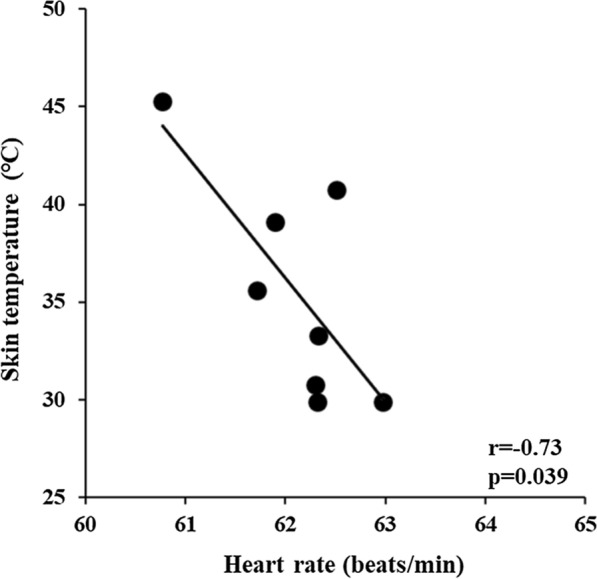
Relationship between heart rate and skin temperature responses during the entire experimental period. The data used in analysis was the mean values per minute averaged over 8 min of the experimental period obtained from for all subjects (*n* = 15)

Table 1 presents the results of heart rate, HRV, blood pressure, and skin temperature. ANOVA revealed a significant task × condition interaction for heart rate, RMSSD, and skin temperature, indicating stronger bradycardia and vagal tone under heat stimulation condition induced by indirect moxibustion than under resting condition. A significant negative correlation between skin temperature and heart rate was also observed (*r* = – 0.73, *p* < 0.05), as shown in Fig. 2. On the other hand, ANOVA revealed no task × condition interaction for blood pressure.

**Table 1:** Heart rate, heart rate variability, blood pressure measured in control (non-burning moxa) and heat stimulation (burning moxa) tasks

Task	Control		Heat stimulation		ANOVA *F-values*		
Condition	Rest	Moxibustion	Rest	Moxibustion	Task x condition	Task	Condition
Heart rate (beats/min)	61.8 ± 8.0	61.8 ± 8.1	63.0 ± 7.8	60.8 ± 7.8	7.6^*^	0.1	7.3^*^
RMSSD (ms)	47.1 ± 21.0	46.5 ± 17.2	52.0 ± 27.1	64.3 ± 37.2	5.2^*^	1.6	3.2
Blood pressure (mmHg)	76.5 ± 11.4	77.1 ± 12.9	79.0 ± 9.6	81.2 ± 11.4	1.7	2.0	2.0
Skin temperature ( ℃)	29.0 ± 1.7	29.6 ± 1.4	29.9 ± 1.4	45.3 ± 3.3	189.4^***^	400.7^***^	311.2^***^

^*^p < 0.05

^***^p < 0.001

The values are the mean and standard deviations for all subjects (*n* = 15)

*RMSSD* root mean square of differences between successive normal R–R intervals. Task: control, heat stimulation. Condition: rest, moxibustion

## Discussion

The present study demonstrated the effects of regional heat stimulation at the acupoint ST36 by indirect moxibustion on heart rate response. A significant bradycardic response was observed when local skin temperature reached a peak by indirect moxibustion. From the results of HRV, modulation of parasympathetic nervous activity was also involved in this response.

Local heat stimulation elicits cardiovascular responses. Yasui et al. [5] demonstrated increased parasympathetic nervous activity and decreased sympathetic nervous activity by applying a heat- and steam-generating sheet on the neck. Nagashima et al. [6] indicated that parasympathetic nerve activity increased following application of a heat- and steam- generating sheet to the abdominal or the lumbar regions. They also observed that application of the heat- and steam- generating sheet improved peripheral hemodynamics and autonomic regulation as demonstrated by significant increases in total hemoglobin and heart rate component on electrocardiogram, inducing a feeling of comfort in the abdomen [11]. Although the area of heat stimulation in the present study was considerably small compared to the sheet used in the previous studies, we observed bradycardia induced by the increase of vagal tone during regional heat stimulation by indirect moxibustion. The heart rate is regulated by the vagal and sympathetic systems. In the present study, indirect moxibustion induced an increase in vagal tone, but sympathetic activity was not observed based on the HRV results. Takahashi et al. [12] also reported a reduction in muscle sympathetic nerve activity associated with a prolonged increase in leg blood flow during local heat application to the skin. Therefore, attenuated sympathetic activity and increased peripheral circulation induced by indirect modulation may contribute to bradycardic response.

On the other hand, there was no significant difference in blood pressure changes between resting condition and moxibustion condition. The vasculature is mainly controlled by sympathetic activity, while blood pressure is regulated by vascular properties including arterial resistance and stressed blood volume [13]. Since indirect moxibustion did not activate sympathetic nerve activity in the present study, consequently vascular properties were probably not modulated resulting in no changes in blood pressure. Future studies are required to answer these questions.

In the present study, heat applied to the acupoint ST36 in the lower leg stimulated a bradycardic response by modulating the autonomic nervous system. Previous studies also reported changes in autonomic nervous activity induced by heat stimulation of the eye, neck, facial, lumber, and abdominal regions [4–7]. These previous studies suggested that thermotherapy elicited autonomic responses regardless of the region of the body stimulated. In the present study, heat stimulation was applied to s small area at the anterior aspect of the leg. Therefore, the change in peripheral blood flow elicited by heat stimulation may be smaller compared to other thermal stimuli such as heat- and steam-generating sheets in previous studies [4–7]. The duration of heat stimulation by indirect moxibustion is also short compared to thermotherapies described in previous studies. Our study thus provides further data of autonomic nervous activity induced by short-duration heat stimulation to a small target region.

Members of the transient receptor potential cation channel family, especially TRPV1, are heat-sensitive ion channels in neurons, which are activated at temperatures higher than 42 °C [14, 15]. Guo et al. [16] indicated that acupuncture stimulation modulates sympathoexcitatory cardiovascular reflex responses through peripheral TRPV1. In the animal study conducted by Wu et al. [17], TRPV1 was found to be present in both neuronal and nonneuronal cells in the regions of the acupoints in the hindlimb, such as ST36. Sun et al. [18] also suggested that heat stimulation induced by moxibustion could activate TRPV1 channels in the acupoint tissue. These findings suggest that TRPV1 plays a key role in the bradycardic response to moxibustion. The present study also indicated a bradycardic effect (from 63.0 to 60.8 beats/min) when the mean skin temperature reached 45 °C. Therefore, heat stimulation at temperatures higher than 42 °C by indirect moxibustion may effectively elicit bradycardic response. We also observed that heat stimulation at temperatures lower than 40 °C did not induce the bradycardic effect and a negative correlation was observed skin temperature and heart rate (Figs. 1, 2). These results suggest that there is temperature-dependence of heat-stimulated heart rate response. As mentioned above, Nagashima et al. [6] observed that applying a heat- and steam-sheet to the abdomen region for 60 min to increase the local temperature to higher than 38.5 °C induced an increase in peripheral blood flow and parasympathetic activity predominance. They also suggested that the heat stimulation could induce some transient receptor potential channels such as TRPV 3 (> 32–39 °C) and TRPV 4 (> 27–35 °C) to exert physiological effects. The bradycardic response started when skin temperature was above 30 °C in the present study (Fig. 2). Therefore, these channels could also play a role in the bradycardic response in the present study. In our study, heat stimulation was applied to the lower leg for 5 min. Factors such as stimulation duration and/or site of stimulation may influence the cardiovascular effects of heat stimulation. In addition, 25% of the subjects are female in the study and the study did not control the menstrual cycle. The previous study indeed indicated that the menstrual cycle altered autonomic nervous system [19]. Further studies on humans are required to elucidate the effects of heat conditions of moxibustion on the cardiovascular responses.

## Conclusion

Regional heat stimulation by indirect moxibustion induces bradycardic response, which is modulated by increased parasympathetic nervous activity. The results provide research evidence that heating by moxibustion stimulates bradycardic response in humans.

## Data Availability

The support data of the present study is available from the corresponding author upon reasonable request.
